# An *In Silico* Platform to Predict Cardiotoxicity Risk of Anti-tumor Drug Combination with hiPSC-CMs Based *In Vitro* Study

**DOI:** 10.1007/s11095-023-03644-4

**Published:** 2023-12-26

**Authors:** Lan Sang, Zhengying Zhou, Shizheng Luo, Yicui Zhang, Hongjie Qian, Ying Zhou, Hua He, Kun Hao

**Affiliations:** 1 State Key Laboratory of Natural Medicines, Jiangsu Province Key Laboratory of Drug Metabolism and Pharmacokinetics, China Pharmaceutical University, Nanjing, 210009 China; 2 Center of Drug Metabolism and Pharmacokinetics, China Pharmaceutical University, Nanjing, 210009 China; 3 School of Life Science and Technology, China Pharmaceutical University, Nanjing, 210009 China; 4 Department of Pharmacy, The First Affiliated Hospital of Nanchang University, Nanchang, 330006 China

**Keywords:** cancer therapy, cardiac toxicity, *in vitro* to *in vivo* translation, pluripotent stem cells, quantitative systems pharmacology

## Abstract

**Objective:**

Antineoplastic agent-induced systolic dysfunction is a major reason for interruption of anticancer treatment. Although targeted anticancer agents infrequently cause systolic dysfunction, their combinations with chemotherapies remarkably increase the incidence. Human induced pluripotent stem cell-derived cardiomyocytes (hiPSC-CMs) provide a potent *in vitro* model to assess cardiovascular safety. However, quantitatively predicting the reduction of ejection fraction based on hiPSC-CMs is challenging due to the absence of the body's regulatory response to cardiomyocyte injury.

**Methods:**

Here, we developed and validated an *in vitro-in vivo* translational platform to assess the reduction of ejection fraction induced by antineoplastic drugs based on hiPSC-CMs. The translational platform integrates drug exposure, drug-cardiomyocyte interaction, and systemic response. The drug-cardiomyocyte interaction was implemented as a mechanism-based toxicodynamic (TD) model, which was then integrated into a quantitative system pharmacology-physiological-based pharmacokinetics (QSP-PBPK) model to form a complete translational platform. The platform was validated by comparing the model-predicted and clinically observed incidence of doxorubicin and trastuzumab-induced systolic dysfunction.

**Results:**

A total of 33,418 virtual patients were incorporated to receive doxorubicin and trastuzumab alone or in combination. For doxorubicin, the QSP-PBPK-TD model successfully captured the overall trend of systolic dysfunction incidences against the cumulative doses. For trastuzumab, the predicted incidence interval was 0.31–2.7% for single-agent treatment and 0.15–10% for trastuzumab-doxorubicin sequential treatment, covering the observations in clinical reports (0.50–1.0% and 1.5–8.3%, respectively).

**Conclusions:**

In conclusion, the *in vitro-in vivo* translational platform is capable of predicting systolic dysfunction incidence almost merely depend on hiPSC-CMs, which could facilitate optimizing the treatment protocol of antineoplastic agents.

**Supplementary Information:**

The online version contains supplementary material available at 10.1007/s11095-023-03644-4.

## Introduction

The constant advancement of antineoplastic drugs and drug administration protocols have significantly prolonged the lifespan of cancer patients, leading to a rapid expansion in the population of cancer survivors. However, antineoplastic drug-induced systolic dysfunction, typically characterized as a reduction in left ventricular ejection fraction (LVEF), has become a primary reason for interrupting anticancer therapy [1]. Such interruptions can result in disease recurrence and a poor prognosis for cancer survivors. Despite the limited incidence of reported cardiac adverse events associated with targeted anticancer agents, their interactions with other chemotherapeutic agents can markedly increase the occurrence of systolic dysfunction. For example, trastuzumab, the first clinically used monoclonal antibody targeting ErbB-2, rarely causes systolic dysfunction when used alone (0.50–1.0%) [2–5]. However, the risk of systolic dysfunction significantly rises when trastuzumab is used concurrently with anthracyclines, such as doxorubicin (11–33%) [6–12] or epirubicin (7.0–56%) [13–16]. Early clinical trials have struggled to identify the relationship between cardiotoxicity and antineoplastic drug combinations due to the exclusion of high-risk patients from recruitment [17], and the limited investigation of complex drug combinations. Therefore, there is an urgent need for methods that can predict the risk of impaired cardiac pumping function associated with antineoplastic agent interactions in humans. Such predictions would enable the optimization of treatment protocols and the reduction of treatment interruptions.

Human-induced pluripotent stem cell-derived cardiomyocytes (hiPSC-CMs) offer a method for predicting drug-induced cardiotoxicity in humans that cannot be captured in preclinical animal studies [18, 19]. In this approach, patient-derived hiPSCs are generated by reprogramming somatic cells, and these hiPSCs are subsequently differentiated into cardiomyocytes. The resulting hiPSC-CMs preserve contractility and can mimic drug-induced changes in morphology and contractile function [20]. In a study by Burridge *et al*., patient-derived hiPSC-CMs successfully replicated the susceptibility of multiple cancer patients to doxorubicin-induced cardiotoxicity [21]. This approach may provide a powerful *in-vitro* method for screening and assessing the cardiovascular safety of antineoplastic drugs, both alone and in combination [18, 22, 23].

Despite the advantages of this approach, *in-vitro* studies of hiPSC-CMs cannot replicate the systemic responses of noncardiac tissues or the pharmacokinetics of the drug. This hinders the translation of the findings of *in-vitro* hiPSC-CMs into a clear understanding of *in-vivo* cardiotoxicity. Mathematical models have been developed to extrapolate *in-vivo* risks by integrating the *in-vitro* findings with additional data on drug exposure and pathophysiology. Successful models using this translational approach include the Comprehensive *in vitro* Proarrhythmia Assay (CiPA) and DILIsym®. CiPA was developed to assess the impact of QTc-prolonging drugs on the risk of torsade de pointes (TdP) based on an *in-vitro* determination of the drug’s effects on multiple ion channels [24–26]. DILIsym® was developed to predict the risk of liver damage by potentially hepatotoxic agents [27–31]. In these models, drug exposure, drug–cell interactions, and consequent systemic responses to alterations in cell structure and function are integrated to predict drug-induced toxicity.

In our previous study, we developed a quantitative system pharmacology (QSP)-physiological-based pharmacokinetics (PBPK)-toxicodynamic (TD) model to predict the reduction of LVEF based on animal studies [32]. The QSP-PBPK-TD model comprises a PBPK model that predicts drug exposure in CMs, an empirical E_max_ model that quantifies the relationship between drug exposure and cardiac injury, and a QSP model that describes the systemic response to cardiac injury after doxorubicin treatment. The hiPSC-CM *in-vitro* approach allows for exploring drug–cardiomyocyte interactions and antineoplastic drug interactions. These *in-vitro* findings can then be quantified using a mechanistic TD model. Integrating the TD model into our previous QSP-PBPK model could facilitate the translation of *in-vitro* experimental observations of hiPSC-CMs into predictions of the incidence of *in-vivo* systolic dysfunction.

Hence, the aim of the present study is to establish a platform capable of translating *in-vitro* findings using hiPSC-CMs to *in-vivo* predictions of the risk of systolic dysfunction due to antineoplastic drugs. This translational platform uses a QSP-PBPK-TD model to integrate drug exposure, drug–cardiomyocyte interactions, and systemic responses to cardiac injury. The QSP-PBPK-TD platform was developed using hiPSC-CMs based on the findings for doxorubicin and trastuzumab treatments given alone, sequentially, or concurrently. We validated the platform by comparing the model’s predictions to experimental findings using hiPSC-CMs and to clinically observed systolic dysfunction incidence in response to doxorubicin–trastuzumab interactions. This *in-vitro*/*in-vivo* translational platform could help optimize treatment protocols for antineoplastic agents.

## Materials and Methods

### Anti-Tumor Drug-induced Cardiotoxicity Assay using hiPSC-CMs

#### Cell culture

Frozen vials of hiPSC-CMs were purchased from HELP (NovoCell™-Cardiomyocytes, Help, China). The vials were thawed and seeded into T-25 flasks (Corning, USA) in a humidified incubator at 37°C and 5% CO_2_, following the manufacturer’s instructions. The cardiomyocytes (CMs) were cultured in a CM culture medium (Help, China) for 5–6 days until they recovered contractile ability and then seeded onto 24-well plates (Corning, USA) that had been pre-encased in CM plate-laying fluid (Help, China). The CMs were seeded at a density of 2 × 10^5^ cells/well. The hiPSC-CMs were used for a drug toxicity test after they regaining contractile ability.

#### Experimental Design

**Fig. 1:**
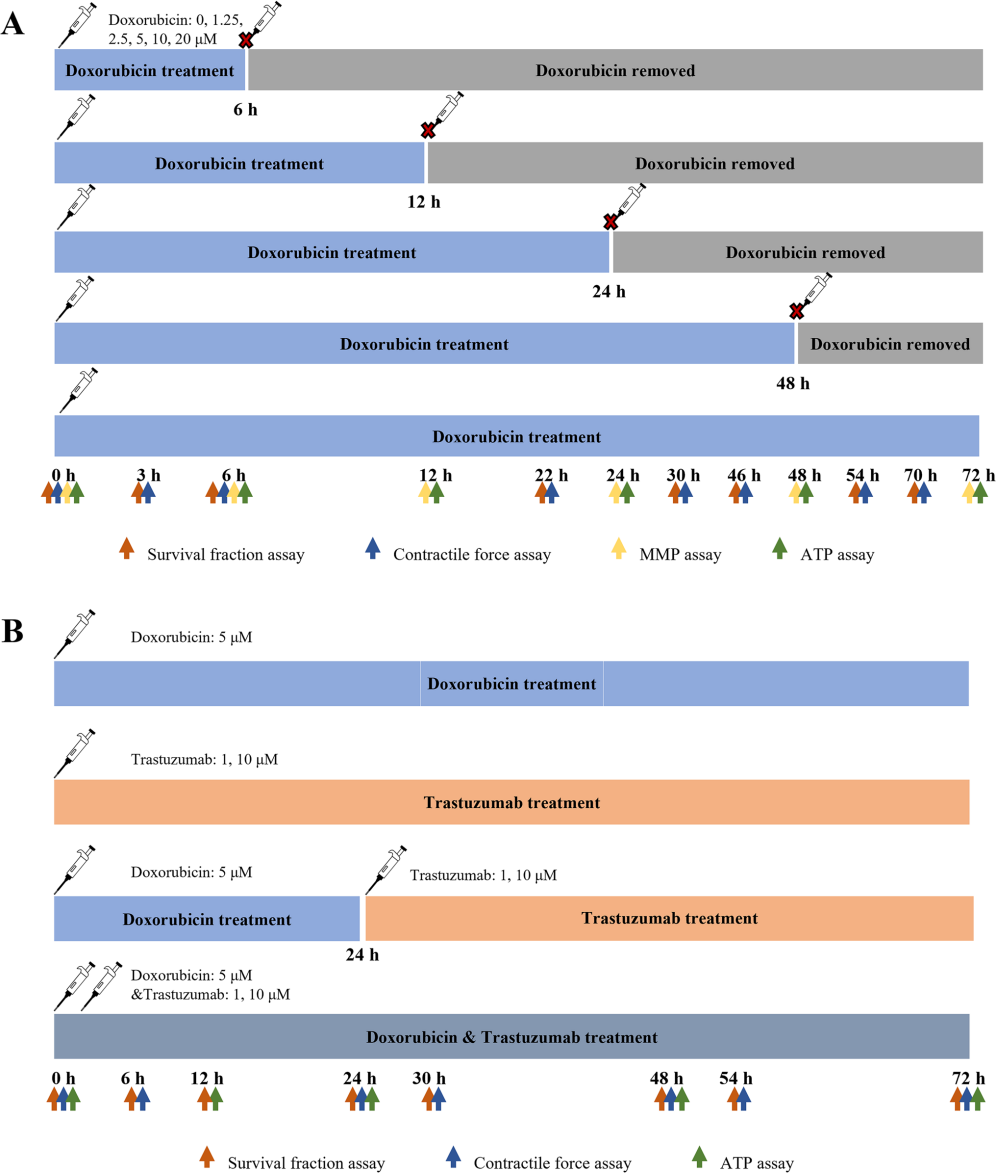
Experimental design for evaluation of drug-induced cardiotoxicity in hiPSC-CMs. (**A**) Influences of different exposure extents (0, 1.25, 2.5, 5, 10, and 20 μM) and durations (6, 12, 24, 48, and 72 h) of doxorubicin on cardiomyocyte contractility. (**B**) Doxorubicin-trastuzumab interaction on cardiomyocyte contractility. Doxorubicin (5 μM) and trastuzumab (1 μM or 10 μM) were given alone, sequentially, or concurrently.

As shown in Fig. 1, the experiment in this study comprised two parts, both using hiPSC-CMs. We examined the impact of exposing hiPSC-CMs to doxorubicin in different amounts (1.25–20 μM) and for different durations (6–72 h), as illustrated in Fig. 1A. After exposure to doxorubicin for the defined time, the cells were washed and cultured in a drug-free CM culture medium until 72 h from the start of the experiment. The remaining survival fraction, CM contractile force, intracellular adenosine triphosphate (ATP) content, and mitochondrial membrane potential (MMP) levels were measured before and 3–72 h after doxorubicin exposure. In the doxorubicin–trastuzumab interaction assay, doxorubicin (5 μM) and trastuzumab (1 μM or 10 μM) were administered alone or in combination (sequentially or concurrently) (Fig. 1B). The remaining survival fraction, CM contractile force, and intracellular ATP content were measured before and 6–72 h after drug exposure.

#### Survival Fraction Assay

The cell survival fraction of cultured hiPSC-CMs was measured to calculate the survival fraction at different timepoints. Cell survival fraction was determined by measuring the cell areas through pixel particle counts at multiple time points in the same well. Three fields of view (upper left, middle, and lower right) in one well were captured using Cytation™ 5 (Bio Tek, USA) and were processed using Image J to determine the fraction of surviving cells.

#### Contractile Force Assay

The average contraction of the CMs was assessed through video microscopy using Cytation^TM^5 (Bio Tek, USA). A 20-s beating profile of the hiPSC-CMs was video recorded at a rate of ten frames per second. The recordings were made at a series of time points, as shown in Fig. 1. The videos were then uploaded to Pulse (www.pulsevideoanalysis.com) for contraction analysis [33]. In our study, the peak contraction magnitude was used to represent CM contraction.

#### ATP Assay

To measure ATP, the CMs were fully lysed using a lysis buffer, and the collected lysate was centrifuged at 12,000 g and 4℃ for 5 min. ATP levels were assessed using the chemiluminescence method in accordance with the manufacturer’s instructions (ATP Assay Kit, Beyotime, China). Luminescence signals were measured using Cytation^TM^5. The protein concentration was measured using the enhanced BCA Protein Assay Kit (Yeasen, China) with a multifunctional microplate reader (Molecular Devices, USA) to obtain the luminescence.

#### MMP Assay

To measure MMP, adherent CMs were first resuspended in each well. Next, 0.5 mL JC-1 staining working solution (Beyotime, China) was added to the resuspended cell solution. The mixture was incubated at 37℃ for 20 min in a cell culture incubator (Thermo, USA) and then centrifuged at 600 g for 3 min. After removing the supernatant, the cells were washed twice and resuspended in 1 mL JC-1 staining buffer. The fluorescence intensity of the suspended cells was measured using Cytation^TM^5. The excitation and emission wavelengths were set to 490 nm and 530 nm to detect JC-1 monomers and to 525 nm and 590 nm to detect JC-1 polymers. MMP levels were calculated using the ratio of polymers to monomers.

#### Data Analysis

All the measurements for survival fraction, ATP, MMP, and contractile force were normalized to baseline. The measurements at each time point were first normalized to the untreated CMs to eliminate inter-well variability and then further normalized to the control CMs at the same time point to account for the impact of circadian rhythm.

### Mechanistic TD Model for Anti-tumor Drug-induced Cardiotoxicity

#### Model Assumptions

**Fig. 2:**
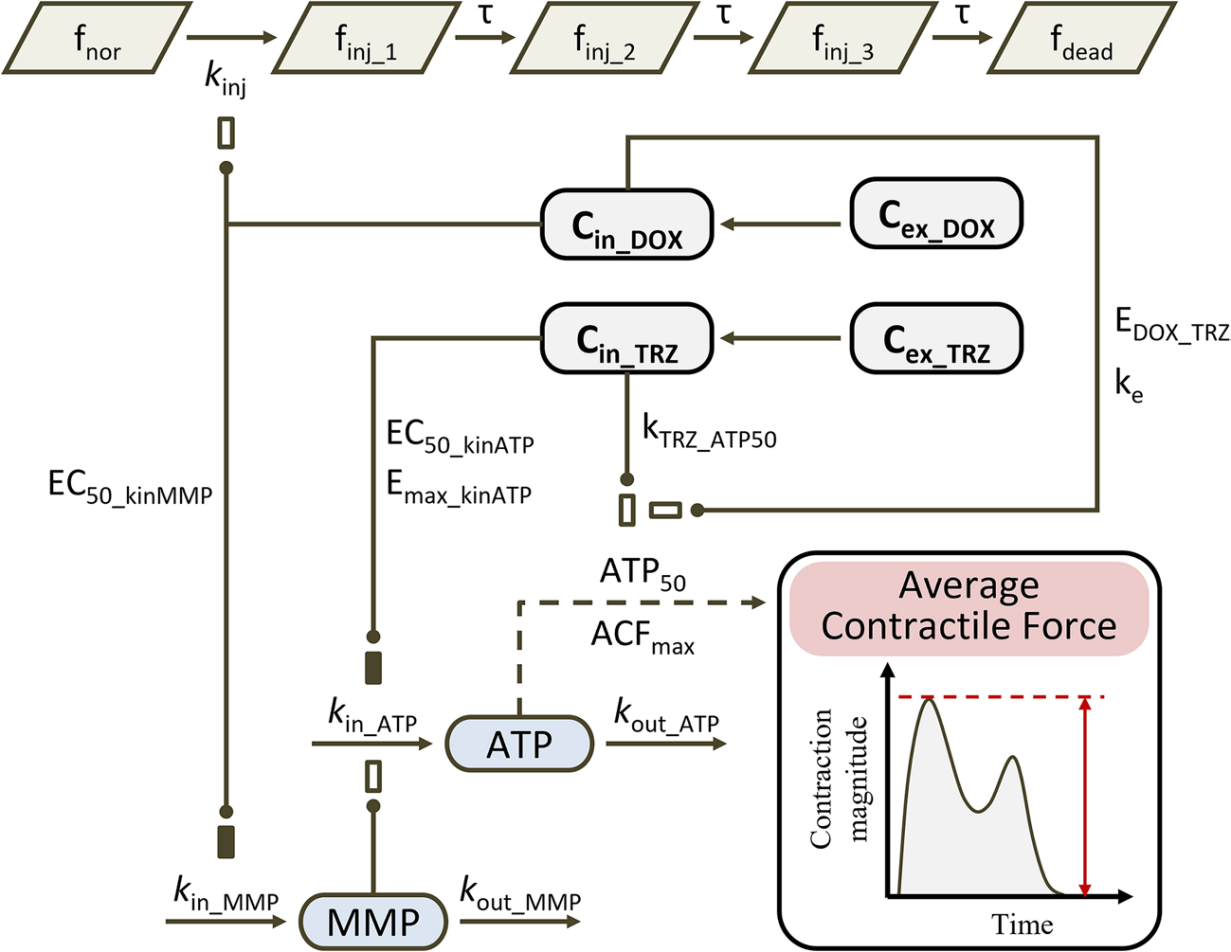
The diagram of potential TD models for drug induced systolic dysfunction. Lines ending in closed circles indicate an effect is being exerted. Open and solid boxes differentiate between stimulatory and inhibitory effects. Dashed lines indicate that arrow-pointed physiological parameters can be calculated by the amount at the other end of lines.

Antineoplastic drugs alter the contractile function of CMs by inducing cell injury or death, inhibiting bioenergy production, or compromising myofibrils. Myocyte injury is an irreversible process caused by a series of events [34]. Therefore, all CMs in the *in-vitro* culture system were divided into three populations according to contractile force: normal, injured, and dead. Normal and injured populations were considered to be the survival population. Furthermore, bioenergy production is the force that drives CM contraction [35]. Hence, in the present model, the contractile force was assumed to be determined primarily by the ATP levels in the cytosol; ATP is generated primarily in the mitochondria. We also assumed that MMP is the primary regulator of the production of high-energy phosphates, including ATP. The chemical energy stored in ATP is converted to mechanical energy through ATP hydrolysis during cross-bridge cycling. The ATPase that participates in this process might be a toxicity target of antineoplastic drugs, and we assumed that this is the mechanism underlying compromised chemo-mechanical energy transduction (Fig. 2). The symbols and parameters used in the model are defined in Table S1.

#### Cellular Drug Exposure

In the present study, intracellular concentration of free anti-tumor doxorubicin (${C}_{in\_DOX}$) were assumed to drive CM dysfunction. A cellular pharmacokinetic model incorporating the drug influx, efflux, and DNA binding was utilized to depict the free doxorubicin profile in culture medium (Eq. 1) and in the cytosol (Eq. 2) [36].

1 $${V}_{cell}\times \frac{{dC}_{in\_total}}{dt}=PER\times {S}_{cell}\times {C}_{ex\_DOX}-\frac{PER\times {S}_{cell}\times {C}_{in\_DOX}}{{k}_{pp}}$$

2 $${C}_{in\_DOX}=0.5\times (({C}_{in\_total}-CN-{k}_{d})+\sqrt{{\left({C}_{in\_total}-CN-{k}_{d}\right)}^{2}+4\times {k}_{d}\times {C}_{in\_total}} )$$

Here, ${V}_{cell}$ is the volume of the CMs; $PER$ is cell membrane permeability; ${S}_{cell}$ is the surface area of a single cell; ${C}_{in\_total}$ is total intracellular doxorubicin concentration; ${C}_{ex\_DOX}$ and ${C}_{in\_DOX}$ are the free doxorubicin concentration in the extracellular culture medium and the intracellular fluid, respectively; ${k}_{pp}$ is the unbound ratio of intracellular and extracellular doxorubicin distribution, based on pH; $CN$ is the concentration of the DNA-binding domain; and ${k}_{d}$ is the equilibrium dissociation constant for drug–DNA binding.

Trastuzumab in cytosol (${C}_{in\_TRZ}$) is the effective concentration and is calculated using Eqs. 3–5 [37].

3 $$\frac{{dC}_{ex\_TRZ}}{dt}={-k}_{on}^{TRZ}\times {C}_{ex\_TRZ}\times \left({Ag}_{cell}-{C}_{bind\_TRZ}\right)+{k}_{off}^{TRZ}\times {C}_{bind\_TRZ}-{k}_{dec}^{TRZ}\times {C}_{ex\_TRZ}$$

4 $$\frac{{dC}_{bind\_TRZ}}{dt}={k}_{on}^{TRZ}\times {C}_{ex\_TRZ}\times \left({Ag}_{cell}-{C}_{bind\_TRZ}\right)-{k}_{off}^{TRZ}\times {C}_{bind\_TRZ}-{k}_{int}^{TRZ}\times {C}_{bind\_TRZ}$$

5 $$\frac{{dC}_{in\_TRZ}}{dt}={k}_{int}^{TRZ}\times {TRZ}_{bound}-{k}_{deg}^{TRZ}\times {C}_{in\_TRZ}$$

Here, ${k}_{on}^{TRZ}$ and ${k}_{off}^{TRZ}$ are the association rate constant and dissociation rate constant between trastuzumab and the ErbB-2 antigen; ${k}_{int}^{TRZ}$ is the internalization rate of trastuzumab inside the cell; ${k}_{deg}^{TRZ}$ is the proteasomal degradation rate of trastuzumab in the endosomal space; ${k}_{dec}^{TRZ}$ is the non-specific deconjugation rate of trastuzumab from the extracellular space; ${Ag}_{CM}^{cell}$ is the number of ErbB-2 s on the cell surface in normal CMs; ${C}_{ex\_TRZ}$ and ${C}_{bind\_TRZ}$ are the concentration of free and bound trastuzumab in the medium; and ${C}_{in\_TRZ}$ is the concentration of trastuzumab internalized in the endosomes of the cells.

#### Myocyte Injury or Death

The variation rates of fractions of normal, injured, and dead myocytes are described by Eqs. 6–12. Gradual myocyte injury is depicted using a transitional model that includes three transitional compartments (Eqs. 7–9). The survival fraction is defined as the sum of normal and injured myocytes (Eq. 12).

6 $$\frac{d{f}_{nor}}{dt}=-{k}_{inj}\cdot {C}_{in\_DOX}\cdot {f}_{nor}$$

7 $$\frac{d{f}_{inj\_1}}{dt}={k}_{inj}\cdot {C}_{in\_DOX}\cdot {f}_{nor}-\frac{{f}_{inj\_1}}{\tau }$$

8 $$\frac{d{f}_{inj\_2}}{dt}=\frac{{f}_{inj\_1}}{\tau }-\frac{{f}_{inj\_2}}{\tau }$$

9 $$\frac{d{f}_{inj\_3}}{dt}=\frac{{f}_{inj\_2}}{\tau }-\frac{{f}_{inj\_3}}{\tau }$$

10 $${f}_{inj}= {f}_{inj\_1}+{f}_{inj\_2}+{f}_{inj\_3}$$

11 $$\frac{d{f}_{dead}}{dt}=\frac{{f}_{inj\_3}}{\tau }$$

12 $$Survival\, fraction=\frac{{f}_{nor}+{f}_{inj}}{{f}_{nor}+{f}_{inj}+{f}_{dead}}$$

Here, ${f}_{nor}$, ${f}_{inj}$, and ${f}_{dead}$ are the fractions of normal, injured, and dead CMs, and ${f}_{inj\_1\sim 3}$ is the fraction of injured myocytes in transitional compartments. The initial value of ${f}_{nor}$ was manually set to 1, and the initial values of ${f}_{inj\_1\sim 3}$ and ${f}_{dead}$ were manually set to 0. ${k}_{inj}$ is the rate constant for normal cells converting to injured cells, and $\tau$ is the transit time for injured myocytes converting to dead cells.

#### Bioenergy Production

Adenosine triphosphate (ATP) is the primary source of bioenergy in living cells. ATP production is regulated by MMP and can be inhibited by trastuzumab (Eq. 13) [38]. We assumed that MMP generation is a zero-order process that could be inhibited by doxorubicin (Eq. 14).

13 $$\frac{dATP}{dt}={k}_{in\_ATP}\times {\left(\frac{MMP}{{MMP}_{0}}\right)}^{n}\times \left(1-\frac{{{E}_{{\text{max}}\_{\text{kinATP}}}\times C}_{in\_TRZ}}{{C}_{in\_TRZ}+{EC}_{50\_kinATP}}\right)-{k}_{out\_ATP}\times ATP$$

14 $$\frac{dMMP}{dt}={k}_{in\_MMP}\cdot \left(1-\frac{{E}_{{\text{max}}\_{\text{kinMMP}}}\times {C}_{in\_DOX}}{{C}_{in\_DOX}+E{C}_{50\_kinMMP}}\right)-{k}_{out\_MMP}\cdot MMP$$

In these equations, ${k}_{in\_ATP}$ and ${k}_{in\_MMP}$ are the zero-order production rate constants of MMP and ATP, and ${k}_{out\_ATP}$ and ${k}_{out\_MMP}$ are the first-order elimination rate constants of MMP and ATP. MMP_0_ is the baseline level of MMP, which was manually set to 1; $n$ is a positive exponent for the stimulatory effect of MMP on ATP production. ${C}_{in\_TRZ}$ is the intracellular trastuzumab concentration (Eq. 5). ${E}_{{\text{max}}\_{\text{kinATP}}}$ is the maximum effect of trastuzumab on ATP production, and ${EC}_{50\_kinATP}$ is the trastuzumab concentration that causes half the maximal effect on ATP production. ${C}_{in\_DOX}$ is the concentration of intracellular doxorubicin (Eq. 2). ${E}_{{\text{max}}\_{\text{kinMMP}}}$ is the maximal effect of doxorubicin on MMP production; it was manually set to 1. $E{C}_{50\_kinMMP}$ is the doxorubicin concentration that causes half the maximal effect on MMP production.

#### Chemo-Mechanical Energy Transduction

An Emax model was used to depict the relationship between the average contractile force (ACF) and the remaining fraction of ATP (Eq. 15). Here, the level of ATP that causes half the maximal ACF (${ATP}_{50}$), was elevated by trastuzumab (Eq. 16). Furthermore, doxorubicin further enhanced trastuzumab’s elevation of ${ATP}_{50}$ (Eqs. 16–17).

15 $$ACF=\frac{{ACF}_{{\text{max}}}\times ATP}{ATP+{ATP}_{50}}$$

16 $${ATP}_{50}= {ATP}_{50\_ref}\times (1+{C}_{in\_TRZ}\times \frac{{k}_{TRZ\_ATP50}}{{E}_{DOX\_TRZ}})$$

17 $$\frac{{dE}_{DOX\_TRZ}}{dt}=-{k}_{e}\times {C}_{in\_DOX}\times {E}_{DOX\_TRZ}$$

In these equations, ${ACF}_{{\text{max}}}$ is the average maximum contractile force that CMs could exert in *in-vitro* experiments (S1 Fig). ${ATP}_{50}$ is the ATP level that caused half the maximum ACF (S1 Fig); ${k}_{TRZ\_ATP50}$ is a linear coefficient for trastuzumab. ${E}_{DOX\_TRZ}$ is doxorubicin’s enhancement of trastuzumab’s effect on ${ATP}_{50}$; the initial value of ${E}_{DOX\_TRZ}$ was manually set to 1 before doxorubicin treatment. ${k}_{e}$ is the efficacy rate constant for doxorubicin’s effect on ${ATP}_{50}$.

#### Parameter Estimation

This study uses a mechanistic TD model to quantify anti-tumor drug-induced cardiotoxicity. The parameters in the TD model were optimized by fitting them to the varying levels of ACF, survival fractions, MMP levels, and ATP content obtained from the *in-vitro* hiPSC-CMs assays. Initial values of system-dependent parameters, including ${k}_{in\_ATP}$, ${k}_{in\_MMP}$, $CN$, and $n$, were initially optimized using the data for doxorubicin. The final values of the system-dependent parameters and the individual-dependent parameters (${k}_{inj}$, $\tau$, and $E{C}_{50\_kinMMP}$) were simultaneously estimated using the trastuzumab–doxorubicin interaction data.

### Use of the QSP-PBPK-TD Model to Translate *in-vitro *Findings to *in-vivo* Predictions

**Fig. 3:**
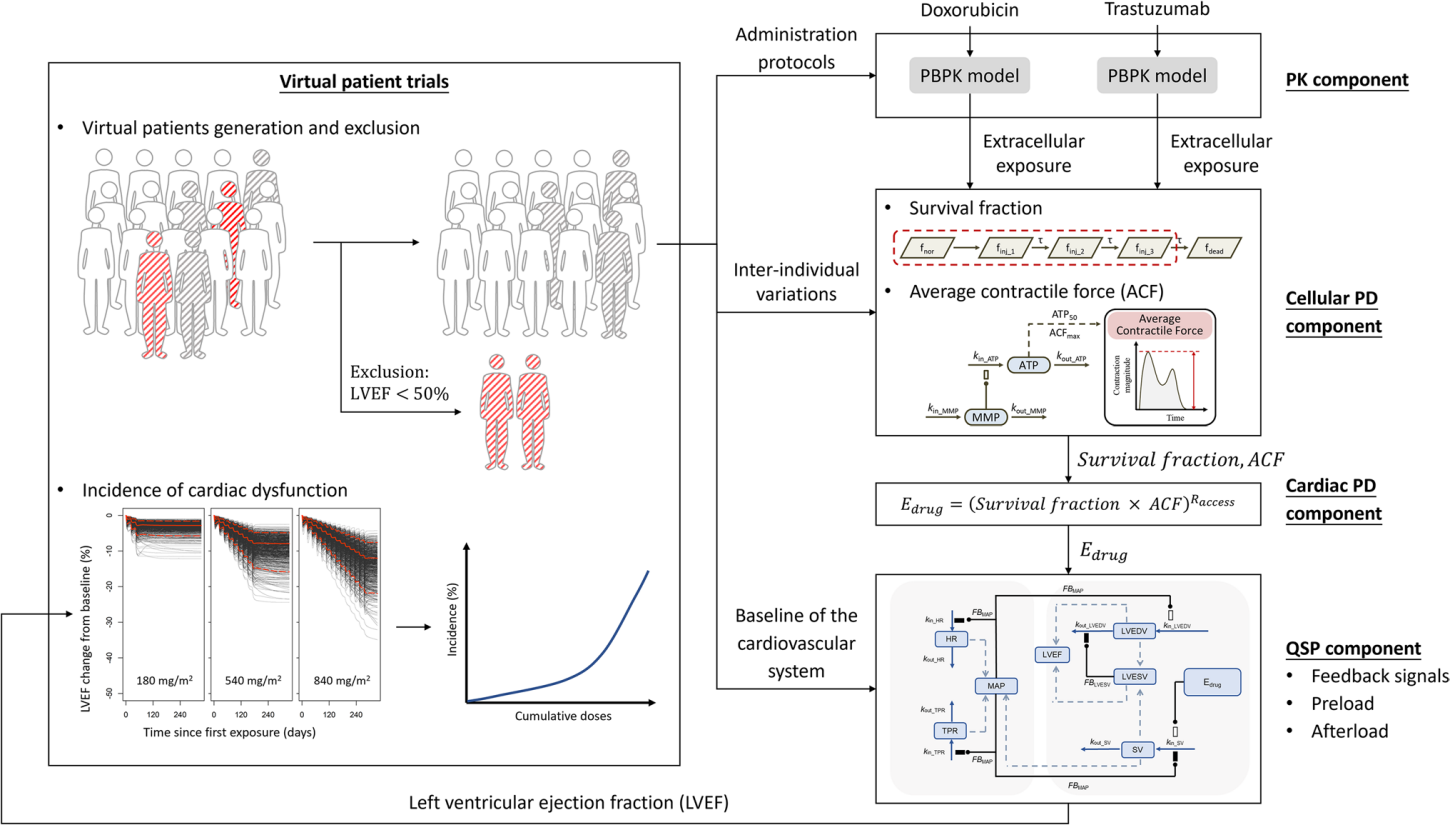
Overall structure of the *in vitro*-*in vivo* translational platform. The platform has four components. In the pharmacokinetic (PK) component, PBPK models convert the doses of antineoplastic agents to their free concentrations in the cytosol. In the mechanism-based toxicodynamic (TD) component, antineoplastic agent interaction and drug-cardiomyocyte interaction jointly determine the variations in survival fraction and average contractile force. The quantitative system pharmacology (QSP) component describes systemic responses to cardiomyocyte injury. Virtual patient trials were conducted to simulate and calculate systolic dysfunction incidence.

The *in vitro*-*in vivo* translation workflow is summarized in Fig. 3. The platform has four components, namely the pharmacokinetic (PK) component, the mechanism-based toxicodynamic (TD) component, the quantitative system pharmacology (QSP) component, and the virtual patient trials. Firstly, drug exposure at the target site was generated by PBPK models in the PK component. Next, antineoplastic agent interaction and drug-cardiomyocyte interaction were obtained from *in-vitro* experiments and were used to modify the parameters. The time-varying survival fraction and the average contractile force (ACF) were then simulated and input into the QSP component, integrating the systemic responses to predict the in vivo impact of drugs on cardiac function. Finally, virtual patient trials were conducted to simulate and calculate the incidence of LVEF reduction.

#### Drug Exposure

In the present study, the PBPK models and the parameters used for doxorubicin [36] and trastuzumab [39] were taken directly from the literature. Extracellular concentrations of antineoplastic agents in the heart were calculated using these PBPK models.

#### Drug–Cardiomyocyte Interaction

The mechanism-based TD model developed in the present study represents drug–cell interactions. In this TD model, the effects of the drugs are driven by the intracellular concentrations of antineoplastic agents, which were calculated based on extracellular concentrations as described in Eqs. 1-5. The joint effects of the drugs on survival fractions and average contractile force were estimated separately, as described in Eqs. 6-12 and 13-17. The drug effects on myocardial contraction in the previous QSP model was modified using the product of the fraction of surviving CMs and the average contractile force estimated by the mechanism-based TD model (Eq. 18).

18 $${E}_{drug}={(Survival\, fraction \times ACF)}^{{R}_{access}}$$

In this equation, ${R}_{access}$ is the ratio of the effective drug concentration in the heart interstitial fluid of that in the cell culture medium. In a 2D cell culture, CMs in the monolayer have equal access to the drug molecules dissolved in the culture medium. However, the 3D structure of the heart means that *in-vivo* CMs have varying levels of access to interstitial drugs. ${R}_{access}$ was therefore introduced to incorporate drug accessibility into the model. The value of ${R}_{access}$ was manually adjusted based on drug concentrations that cause equivalent effects on the fraction of surviving cells in 2D cultured cells or in the heart [40].

#### Systemic Response

A previously developed QSP model was utilized to depict the systemic responses of the body to cardiac injury induced by antineoplastic agents [32]. The drug’s effect on stroke volume (${E}_{EP\_SV}$) was replaced by the effect calculated by our TD model (${E}_{drug}$). The QSP model could be used to simulate LVEF variations in response to antineoplastic treatment.

#### Virtual Patient Trials

We conducted virtual human trials to simulate and calculate the incidence of LVEF reduction. Virtual patients were generated using Monte Carlo sampling. The coefficients of variation that were reported or estimated for parameters in the QSP-PBPK-TD model were incorporated to better replicate the conditions of real patients in terms of drug exposure, systemic regulation, and myocardial susceptibility. Based on clinical measurements of drug-induced cardiotoxicity, we considered a drop in LVEF by more than 10% from baseline or to less than 50% of baseline to indicate LVEF reduction [41].

### Validation of the Method for Translating *in-vitro* Findings to *in-vivo* Predictions

**Fig. 4:**
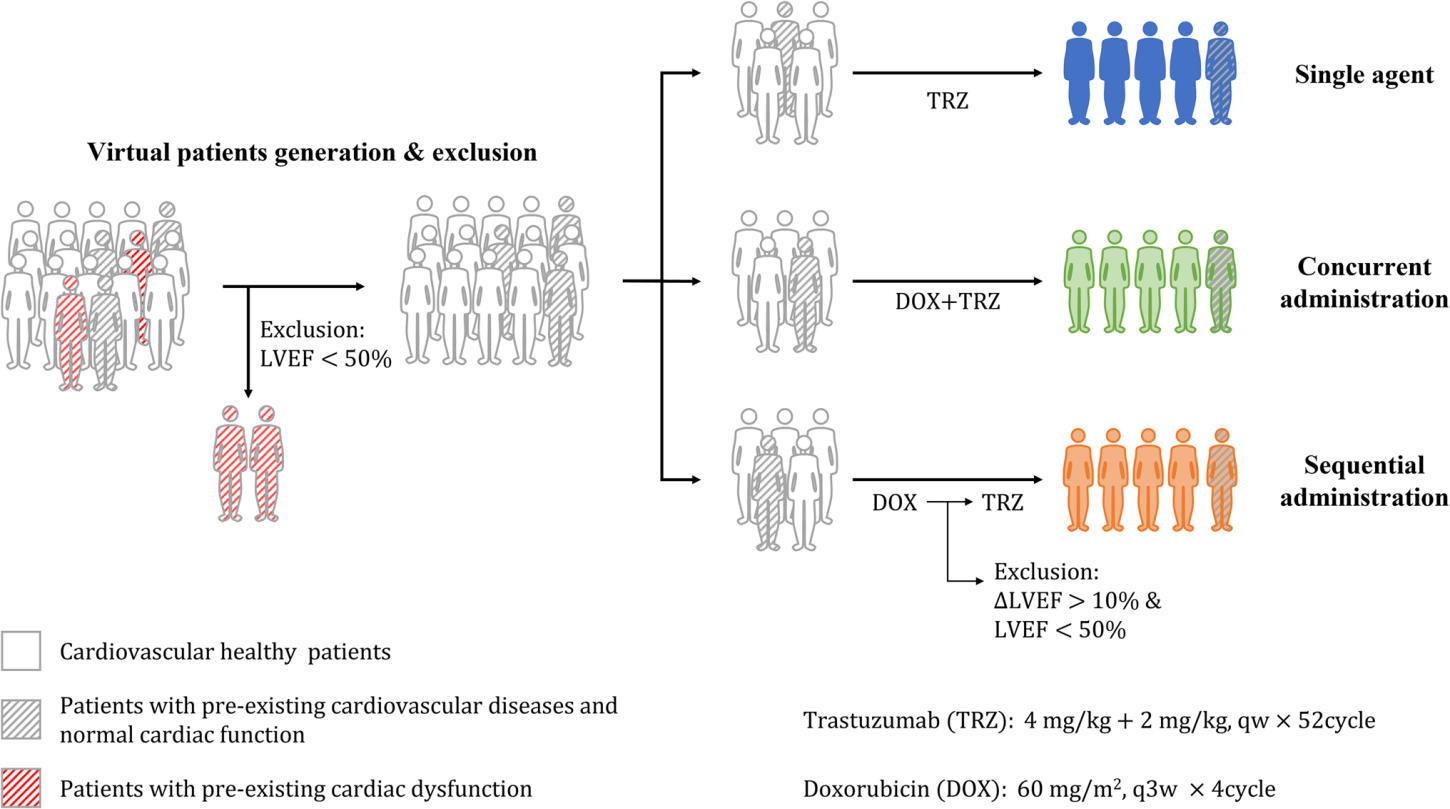
Experimental design for virtual trials. TRZ: single-agent trastuzumab group; DOX → TRZ: sequential administration of doxorubicin and trastuzumab; DOX + TRZ: concurrent administration of doxorubicin and trastuzumab.

Virtual patient trials were conducted to validate our translational approach. For doxorubicin, which is believed to induce dose-dependent cardiotoxicity [42], the rate of incidence of systolic dysfunction after cumulative doses in virtual patients was compared to that reported in clinical trials [43–45]. The doxorubicin administration protocol was based on the recommended clinical regimen (IV infusion of 60 mg/m^2^ every three weeks, with cumulative doses ranging from 60 to 960 mg/m^2^) [46]. The incidence of systolic dysfunction was simulated one year after the first dose of doxorubicin [47]. To measure doxorubicin–trastuzumab interactions, three trastuzumab dosing regimens were included (Fig. 4): (1) Single-agent group (TRZ): virtual patients were treated with 2 mg/kg of trastuzumab weekly for 52 weeks, with an additional dose of 2 mg/kg the first week. (2) Sequential group (DOX → TRZ): virtual patients received 60 mg/m^2^ of doxorubicin every three weeks for 12 weeks; after this, they received 2 mg/kg of trastuzumab weekly for 52 weeks, with an additional dose of 2 mg/kg the first week. (3) Concurrent group (DOX + TRZ): virtual patients received doxorubicin and trastuzumab concurrently, with the same doses and durations as those used for the sequential group [48]. The incidence of systolic dysfunction at week 64 after the first dose was simulated. Virtual patients who developed systolic dysfunction before trastuzumab administration were excluded from the trial [49]. We also investigated the influence of cardiovascular complications on drug-induced cardiotoxicity. Hypertension (mean arterial pressure ≥ 115 mmHg) or dilated cardiomyopathy (left ventricular end-diastolic volume ≥ 146 mL) were considered cardiovascular complications that would compromise the systolic regulation of myocardial injury in patients [50].

### Sensitivity Analysis

A sensitivity analysis was performed to identify the key factors determining anti-tumor drug-induced cardiotoxicity. The incidence of systolic dysfunction after the sequential administration of doxorubicin and trastuzumab (as described above) was used as an indicator of cardiotoxicity. TD parameters, including system-dependent parameters (${k}_{in\_ATP}$, ${k}_{in\_MMP}$, and $n$) and individual-dependent parameters (${k}_{inj}$, $\tau$, $E{C}_{50\_kinMMP}$, ${E}_{{\text{max}}\_{\text{kinATP}}}$, ${EC}_{50\_kinATP}$, ${k}_{TRZ\_ATP50}$, and ${k}_{e}$) were modified from 1/3- to 3 × their original values for the sensitivity analysis. Parameter sensitivities were calculated using Eq. 19.

19 $$Sensitivity= \frac{{p}_{i}}{Incidence}\times \frac{\Delta Incidence}{\Delta {p}_{i}}$$

Here, ${p}_{i}$ is the original value (or the estimated value listed in Table I) for a TD parameter. $Incidence$ is the incidence of systolic dysfunction simulated by the QSP-PBPK-TD model with all the original values of the TD parameters. $\Delta {p}_{i}$ is the change in a TD parameter from its original value. $\Delta Incidence$ is the difference in incidence calculated using the new and original TD parameters.

**Table I:** Summaries of TD Model Parameters

Parameters	Unit	Values	
		DOX (RSE%)	DOX-TRZ interaction (RSE%)
System-dependent			
${k}_{in\_ATP}$	h^-1^	0.032 (4.09)	
${k}_{in\_MMP}$	h^-1^	0.96 (38.7)	
$CN$	μmol/L	7.21×10^4^ (24.3)	
$n$	-	0.8 (8.98)	
Individual-dependent			
${k}_{inj}$	(h$\cdot$μmol/L)^-1^	0.0023 (20.6)	0.0035 (8.47)
$\tau$	h	1.38 (17.9)	4.9 (7.86)
$E{C}_{50\_kinMMP}$	μmol/L	0.36 (18.4)	1.87 (24.8)
${E}_{{\text{max}}\_{\text{kinATP}}}$	-	-	0.54 (22.1)
${EC}_{50\_kinATP}$	nmol/L	-	30.11 (25.3)
${k}_{TRZ\_ATP50}$	(nmol/L)^-1^	-	0.013 (14.8)
${k}_{e}$	(h$\cdot$μmol/L)^-1^	-	9.94 (11.2)

### Software and Hardware

Image J (https://imagej.net) was used to analyze the survival fraction. The website Pulse Video Analysis (www.pulsevideoanalysis.com) was used to analyze the magnitude of contraction. The stochastic approximation expectation–maximization algorithm implemented in Monolix 2019 R2 (https://monolix.lixoft.com) was used to minimize the objective function in the mechanism-based TD model. The model’s goodness-of-fit was tested using the Akaike information criterion (AIC), the Bayesian information criteria (BIC), the corrected BIC (BICc), and the relative standard error (RSE). All simulations were conducted on Berkeley Madonna (https://berkeley-madonna.myshopify.com). Cytation™ 5 (Bio Tek, USA) was used to record the fraction of surviving cells, the contraction videos, and the luminescence signals used to measure ATP and MMP.

## Results

### Doxorubicin- and Trastuzumab-induced Cardiotoxicity in hiPSC-CMs

**Fig. 5:**
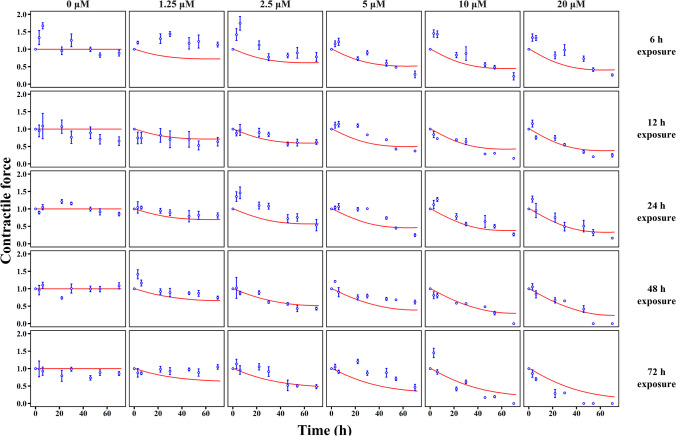
Model predictions (red line) and experimental observations (blue dot) for contractile force in hiPSC-CMs in response to doxorubicin. Red lines indicate model predictions and blue dots indicate experimental observations.

First, we explored the impact of the amount and duration of doxorubicin exposure on contractile function, which was evaluated based on the average contractile force (ACF) of cells in one plate. As shown in Fig. 5, doxorubicin-treated hiPSC-CMs exhibited irreversible concentration-dependent contractile dysfunction. Prolonged exposure amplified this impairment, suggesting that both the amount and duration of exposure impact doxorubicin-induced cardiotoxicity as independent variables. The underlying mechanism of doxorubicin-induced systolic dysfunction was explored by measuring the survival fraction (S2 Fig), intracellular ATP levels (S3 Fig), and MMP levels (S4 Fig). The survival fraction reduced as the amount and duration of drug exposure increased only when the concentration of doxorubicin was greater than 1.25 μM (S2 Fig). As shown in S3 Fig and S4 Fig, ATP and MMP levels decreased as the amount of doxorubicin increased. However, no significant difference was observed in ATP and MMP levels when the duration of exposure was extended from 6 to 72 h. Hence, the amount of doxorubicin exposure is the only independent variable that affects bioenergy production.

**Fig. 6:**
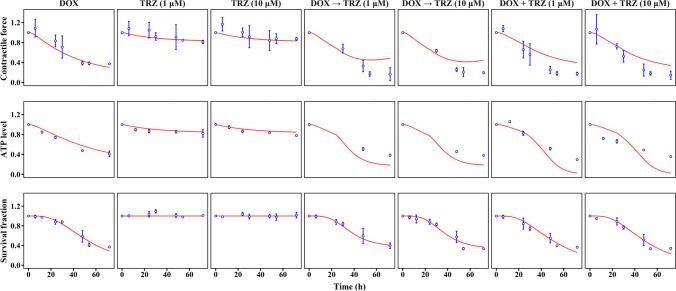
Model predictions and experimental observations of contractile force, survival fraction, and ATP levels in hiPSC-CMs. Red lines indicate model predictions and blue dots indicate experimental observations. Doxorubicin (5 μM) and trastuzumab (1 μM or 10 μM) were given alone, sequentially, or concurrently.

Second, we investigated the impact of the interaction of doxorubicin and trastuzumab on cardiomyocyte contraction (Fig. 6). When trastuzumab was given alone, the hiPSC-CMs showed slight decreases in contractile force and intracellular ATP content, but there was no significant change in the survival fraction. Compared to the trastuzumab-only group, the sequential group showed a 5.4-fold decrease in contractile force and a 3.2-fold decrease in ATP level within 72 h. In addition, the survival fraction decreased by around 60% in the sequential group. The concurrent group showed even more severe contractile dysfunction than the sequential group (ΔACF = 0.44 *vs*. 0.32 at 30 h; ΔACF = 0.75 *vs*. 0.68 at 48 h). No significant difference in survival fraction or ATP level was observed between the sequential group and the concurrent group. Moreover, compared with simply adding doxorubicin and trastuzumab’s effects together, the synergistical toxicity of doxorubicin and trastuzumab (whether given sequentially or concurrently) resulted in an 11% greater inhibition of ACF and a 118% decrease in the survival fraction. This synergistic effect, however, was not observed in ATP levels; the decrease in ATP levels in the combined group was 28% greater than the sum of the decreases in the doxorubicin and trastuzumab groups.

### TD Model for Drug-induced Cardiotoxicity

Figure 5 presents predicted and observed individual data for average contractile force, survival fraction, and bioenergy production of hiPSC-CMs treated with doxorubicin. for hiPSC-CMs treated with trastuzumab alone or in combination with doxorubicin, the the individual predictions and observations are illustrated in Fig. 6. The model parameters can be found in Table I, and detailed explanations for these parameters are provided in Table S1. Overall, the model successfully captures the observed variations in contractile force, survival fraction, and bioenergy production.

The rate constant of cell injury triggered by doxorubicin (${k}_{inj}$) was estimated as 0.0035 h^−1^, suggesting that 0.35% of the remaining myocytes are injured by doxorubicin every hour. The process in which injured cells convert to dead cells is illustrated by a transduction model with three transitional compartments, and the transit time ($\tau$) was estimated to be 4.9 h. This implies that injured myocytes retain their contractile ability for an extended period before eventual cell death. This observation aligns with clinical findings where elevated cyclic troponin levels can be immediately measured after the first dose, [51], yet insufficient systolic dysfunction might not be detected [52]. Additionally, $E{C}_{50\_kinMMP}$, representing the doxorubicin concentration causing half the maximal effect on MMP production, was estimated to be 0.36 μmol/L.

When trastuzumab is administrated alone, it not only interferes with the ATP production rate [38] but also impacts chemo-mechanical energy transduction. The hypothesis is supported by our findings, which reveal that contractile force decreases more than ATP content (Fig. 5). The parameter ${E}_{{\text{max}}\_{\text{kinATP}}}$ was estimated to be 0.54, indicating that trastuzumab could maximally inhibit 54% of ATP production in the current study. The parameter ${EC}_{50\_kinATP}$ and ${k}_{TRZ\_ATP50}$, which reflect CM susceptibility to trastuzumab, are estimated to be 30.11 μg/mL and 0.013 (μg/mL)^−1^, respectively.

When doxorubicin was administered before or in combination with trastuzumab, the susceptibility of CMs to trastuzumab’s impacts on chemo-mechanical energy transduction were upregulated. The coefficient of doxorubicin on trastuzumab’s effect (${k}_{e}$) was estimated as 9.94 (h $\bullet$ μmol/L) ^−1^, indicating a 20% increase in the trastuzumab-induced reduction in contractile force in a steady state. Doxorubicin’s upregulation of myocytes’ susceptibility to trastuzumab might be the underlying mechanism of doxorubicin–trastuzumab interactions, leading to a synergistic cardiotoxic effect.

### *In-vitro *to *in-vivo* Extrapolation of Drug-induced Cardiotoxicity

The 3D structure of *in-vivo* heart tissue reduces CM drug accessibility compared to an *in-vitro* 2D culture. To account for this difference in drug exposure, we introduced the drug accessibility parameter, ${R}_{access}$, which was set to 1 for the *in-vitro* 2D culture. To model the incomplete accessibility of the interstitial drug to CMs in the heart tissue, especially those far from the blood vessel, ${R}_{access}$ was reduced to 0.12 for the *in-vivo* simulation. This adjustment was informed by the observation that the ratio of CM sensitivity to doxorubicin in a 3D iPSC-CM culture is 0.085 lower than that in a 2D culture [40].

**Fig. 7:**
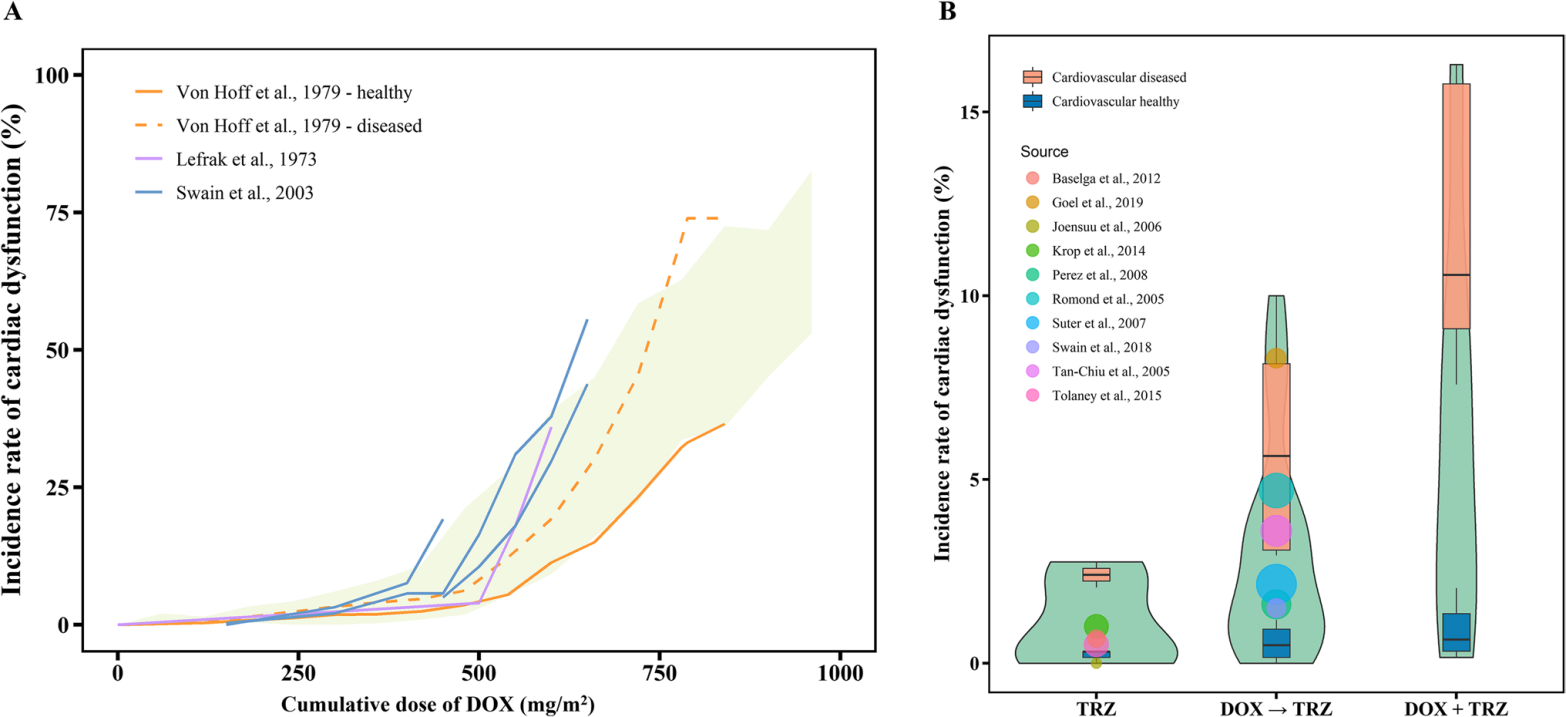
Observed and model predicted incidences of doxorubicin and trastuzumab induced systolic dysfunction. (**A**) Observed and model predicted incidences of doxorubicin-induced systolic dysfunction. The light green shadow represents prediction interval of the incidence. Lines in different colors represent incidence rates reported by different clinical trials [43–45]. Solid and dashed lines represent incidence rates for cardiovascular normal and diseased patients. (**B**) Observed and model predicted incidences of trastuzumab-induced systolic dysfunction [2–5, 49, 53–57]. The violin plots represent the distribution of incidence calculated in all virtual patients. Box plots represent simulated incidence of systolic dysfunction in patients in different cardiovascular conditions. Bubbles in different colors represent observed incidences reported by various clinical trials. TRZ: single-agent trastuzumab treatment; DOX → TRZ: sequential treatment of doxorubicin and trastuzumab; DOX + TRZ: concurrent treatment of doxorubicin and trastuzumab.

The incidence of doxorubicin- and trastuzumab-induced systolic dysfunction was simulated to validate the *in-vitro* to *in-vivo* extrapolation used in this study. Among the 16,000 virtual patients generated by Monte Carlo sampling, 15,939 virtual patients with LVEF ≥ 50% were incorporated into the virtual patient trials, including 5,964 patients with and 9,975 patients without hypertension or dilated cardiomyopathy. As shown in Fig. 7A, the observed cumulative dose–response relationship from three clinical trials falls within the 90% confidence range of the predicted dose–response curve. These results suggest that the translational approach proposed in the current study is able to capture the overall trend of systolic dysfunction caused by doxorubicin.

Cardiotoxicity due to trastuzumab monotherapy and sequential/concurrent treatment with doxorubicin was also simulated to evaluate the *in-vitro* to *in-vivo* extrapolation used here. A total of 18,000 virtual patients were generated via Monte Carlo sampling. Of these, 17,479 virtual patients with LVEF ≥ 50% were incorporated into the virtual patient trials, including 6,415 patients with and 11,064 patients without cardiovascular comorbidity. As illustrated in Fig. 7B, the predicted incidence of systolic dysfunction due to trastuzumab monotherapy ranges from 0.31% to 2.7%, aligning with previous clinical reports (0.50–1.0%) [2–5]. For sequential treatment with doxorubicin and trastuzumab, the predicted incidence of systolic dysfunction ranges from 0.15% to 10%, overlapping with clinical observations (1.5–8.3%) [49, 53–57]. Although there are no clinical data to validate the predicted incidence of concurrent treatment with trastuzumab and doxorubicin, the higher predicted incidence suggests that this schedule involves higher risks. This also aligns with our findings, which suggest that concurrent treatment with these drugs should be avoided. Moreover, the estimated incidence of systolic dysfunction in patients with pre-existing cardiovascular complications is significantly higher than that in patients with normal cardiovascular function, suggesting that cardiovascular disease might be a nonnegligible factor in this risk, as suggested in other studies [43, 50, 58]. These results imply that the current QSP-PBPK-TD model can accurately predict the cardiotoxicity of doxorubicin and trastuzumab under different treatment schedules.

### Sensitivity Analysis

Sensitivity analyses were conducted for parameters reflecting doxorubicin’s toxic effects on cell injury (${k}_{inj}$) and mitochondrial membrane damage ($E{C}_{50\_kinMMP}$). As depicted in S5A and S5B Fig, doxorubicin-induced cardiotoxicity is significantly influenced by ${k}_{inj}$. Furthermore, individuals with pre-existing cardiovascular complications were found to be more susceptible to doxorubicin-induced cardiotoxicity compared to those without cardiovascular comorbidities. For instance, even with the same ${k}_{inj}$ value of 1.15 × 10^–3^ (h $\cdot$ μmol/L) ^−1^, the risk of a patient with pre-existing cardiovascular disease developing doxorubicin-induced systolic dysfunction at a cumulative dose of 480 mg/m^2^ is 4.32%, while this risk is 0.166% for patients with no cardiovascular comorbidities. On the other hand, $E{C}_{50\_kinMMP}$ appears to function as a less sensitive parameter in the TD model. No significant difference in the incidence curves can be observed when the cumulative dose of doxorubicin is less than 720 mg/m^2^. Even at a cumulative dose higher than 720 mg/m^2^, there was no significant difference between the incidence rates when $E{C}_{50\_kinMMP}$ is increased to 3.6 nM from the original value of 0.36 nM. A detectable increase in incidence rates was only observed when $E{C}_{50\_kinMMP}$ was reduced to one-tenth of the original value (0.036 nM) (S5C and S5D Fig).

According to the sensitivity analysis, the coefficients for trastuzumab (${EC}_{50\_kinATP}$, ${k}_{TRZ\_ATP50}$, and ${E}_{{\text{max}}\_{\text{kinATP}}}$) are the most sensitive parameters in the TD model. This indicates that inter-individual variance in patients’ susceptibility to trastuzumab is the factor with the greatest impact on trastuzumab’s cardiotoxicity. The sensitivity of ${EC}_{50\_kinATP}$ was estimated as 19.3, indicating that, under same effective concentration of trastuzumab, a 10% decrease in ${EC}_{50\_kinATP}$ leads to an almost 200% increase in the incidence of systolic dysfunction. The rate constant of cell injury triggered by doxorubicin (${k}_{inj}$) is a sensitive factor as well, indicating that doxorubicin-induced cell death may indirectly impair patients’ tolerance to trastuzumab. However, system-dependent parameters seem to have a less impact on cardiotoxicity compared to individual-dependent parameters (S6 Fig).

## Discussion

Using hiPSC-CMs as an *in-vitro* tool for assessing drug-induced cardiotoxicity, this study has developed a model-based approach for translating *in-vitro* results to *in-vivo* predictions, focusing on antineoplastic agents. The *in-vitro* data were used to develop a mechanistic TD, which was then integrated into the PBPK models for doxorubicin and trastuzumab and into a QSP model describing the systemic response to cardiac injury [32, 36, 39]. Using this *in-vitro* to *in-vivo* translational platform, the systolic dysfunction incidence for doxorubicin and trastuzumab alone or in sequential combination has been predicted and validated by comparing our findings to published clinical results.

The utilization of QSP modeling has significantly advanced our comprehension of complex cardiovascular processes. Notably, it has allowed us to gain insights into various aspects of cardiovascular health. For instance, in the context of peripheral arterial disease, multiscale models have elucidated the dynamic process of perfusion recovery post-ischemia, providing valuable information for potential therapeutic interventions [59]. Additionally, integrated cardiorenal models have provided crucial insights into adaptive cardiac remodeling in response to pressure and volume overload, shedding light on the interplay between cardiac and renal function [60]. Furthermore, QSP modeling has quantitatively assessed the multifaceted effects of sodium-glucose cotransporter 2 (SGLT2) inhibitors on renal hemodynamics, volume status, and blood pressure, enhancing our knowledge of cardiorenal interactions [61]. These applications underscore the potential of QSP modeling in advancing our comprehension of cardiovascular phenomena and guiding clinical practices and treatment strategies.

A recent work published by Fu *et al*. has also employed QSP modeling to quantify inter-relationship between biomarkers in cardiovascular system [62]. In this study, the researchers consider SV as a function of both contractility and heart rate, while we simplify this assumption by focusing solely on contractility's influence on SV. This methodological difference stems from their use of atenolol, a heart rate-affecting drug, while our focus is on trastuzumab and doxorubicin, which currently show no reported impact on heart rate. Additionally, our primary research objective differs from that of Fu *et al*. While their study aims to elucidate the relationship between myocardial contractility and cardiovascular markers, our focus is on predicting the risk of anticancer drug-induced systolic dysfunction. Therefore, the translational conversion of changes in myocardial contractility from *in vitro* to *in vivo* is the key focus of our study.

It is noteworthy that the *in-vitro* to *in-vivo* translational platform is capable of translating cardiotoxicity, involving not only doxorubicin and trastuzumab but also other antineoplastic agents, regardless of their anti-tumor mechanisms. For drug combinations that have not yet been implemented in clinic, the risk of systolic dysfunction could be forecasted by the QSP-PBPK-TD platform by adjusting the individual-dependent parameters in the TD model and the drug-specific PK models. The individual-dependent parameters could be optimized through *in vitro* measurements on hiPSC-CMs without modifying the model structure. This model-based approach is especially beneficial for drugs under development since it could provide predictions before risking any patients, thereby facilitating first-in-human strategies for antineoplastic combination.

In our *in-vitro* study using hiPSC-CMs, doxorubicin-induced cardiotoxicity depended on both the amount and duration of exposure. However, trastuzumab-induced cardiotoxicity was independent of concentration the clinically relevant range of 1 μM to 10 μM. To explore the underlying mechanisms of these observations, we simulated the trastuzumab concentration in the extracellular medium, the concentration in intracellular space, and the concentration binding to the target enzyme (S7 Fig). The results suggest that the ErbB-2 receptor occupancy of trastuzumab reaches its maximum at 1 μM, and higher levels of trastuzumab exposure result in identical receptor occupancy, leading to similar toxic effects.

HiPSC-CMs mimic patient-specific susceptibility to cardiotoxic drugs, resulting in high levels of variation in our *in-vitro* findings. In the current study, we observed that myocardial susceptibility varied among experiments, even when using the same doxorubicin dose (Fig. 5 and 6**,** S2-4 Fig). Although we used the same cell strain (hiPSC-CMs), different differentiation batches of hiPSC might have caused discrepancies in cell susceptibility. These results align with a previous report indicating that hiPSC-CMs derived from different patients exhibit a 20-fold variance in susceptibility to doxorubicin [21]. This variation in myocardial susceptibility makes it challenging to predict drug-induced LVEF reduction in clinical practice. To quantify myocardial susceptibility, we separated individual-dependent parameters from system-dependent parameters. In the present study, system-dependent parameters include the kinetics of energy metabolism (${k}_{in\_ATP}$, ${k}_{in\_MMP}$, and $n$) and non-drug-related protein binding sites ($CN$); these were assumed to be stable across all experiments that used hiPSC-CMs cell lines. Individual-dependent parameters (i.e., ${k}_{inj}$, $\tau$, and $E{C}_{50\_kinMMP}$), determining cellular responses to specific drug strategies, can vary considerably across individuals. Our sensitivity analysis shows that system-dependent parameters exert weaker effects than individual-dependent parameters (S3 Fig). Therefore, we used doxorubicin as an internal standard of cardiotoxic agents to allow our findings to be compared to various *in-vitro* experiments. The effects of doxorubicin on hiPSC-CMs were used to estimate the fixed system-dependent parameters and the initial values of individual-dependent parameters (Table I).

The research revealed the underlying mechanism of clinically synergistic cardiotoxicity associated with sequential doxorubicin and trastuzumab treatment. Two mechanisms explain their interactions [63, 64]. The first suggests that an ErbB-2-dependent recovery phase follows doxorubicin treatment, and trastuzumab inhibits this recovery. The second indicates an ErbB-2-independent mechanism, where trastuzumab itself has an inhibitory effect on contractile components. In the current study, the *in-vitro* measurements did not reveal hiPSC-CMs recovered after doxorubicin exposure (Fig. 5 and 6**, **S2-4 Fig), leading to our inability to precisely estimate the recovery rate of myocardial cells (RSE = 80.7%). Therefore, we integrated the second mechanism into the final model. However, the failure to precisely estimate the recovery rate does not rule out the possibility of this mechanism in doxorubicin–trastuzumab interactions. Future studies should test this mechanism using experimental methods capable of measuring DNA or RNA recovery. Regarding cardiotoxicity induced by doxorubicin–trastuzumab interactions, apart from the coefficients of trastuzumab, ${k}_{inj}$ is the most influential factor. Since cell injury is an irreversible process ultimately leading to cell death, a history of myocyte exposure to doxorubicin may cause cell loss, compromising the heart’s tolerance to toxic agents and consequently increasing the risk of drug-induced systolic dysfunction [65].

Despite the successful extrapolation of *in-vitro* findings to *in-vivo* predicitons, our hiPSC-CM-based translational approach has some limitations. The generated hiPSC-CMs are less mature than CMs in patients’ hearts, potentially impacting the accuracy of our model’s predictions of drug-induced systolic dysfunction [66–68]. Incorporating a maturation process for hiPSC-CMs to simulate more mature CMs in the TD model could enhance predictive accuracy [69]. Moreover, beyond the direct cytotoxic effect on cardiomyocytes, drug-induced systolic dysfunction could result from non-cytotoxic mechanisms, such as a proarrhythmic effect, which our model currently does not consider. To address these limitations, future studies should continue to advance the use of hiPSC-CMs in studying cardiotoxicity and integrate the current approach with other strategies for predicting cardiotoxicity, such as CiPA.

## Conclusion

In summary, we have developed and validated a translational platform that uses hiPSC-CMs to assess antineoplastic drug-induced systolic dysfunction. The translational platform comprises a PBPK model that depicts drug exposure, a TD model that depicts drug–cell interactions, and a QSP model that depicts the resulting systemic response. The TD model developed in this study goes beyond prior models to capture systolic dysfunction by integrating CM injury, bioenergy production, and chemo-mechanical energy transduction. This translational approach is suitable for predicting the incidence of systolic dysfunction induced by antineoplastic agents in drug development, which might facilitate the optimization of antineoplastic agent protocols.

### Supplementary Information

Below is the link to the electronic supplementary material.

Supplementary file1 (DOCX 1298 KB)

## Data Availability

The data generated in this study are available in this article and its supporting information files.
