# Supplementary material for: An In Silico Platform to Predict Cardiotoxicity Risk of Anti-tumor Drug Combination with hiPSC-CMs Based In Vitro Study
Source: Pharm Res. 2023 Dec 26;41(2):247–62. doi: 10.1007/s11095-023-03644-4 (PMC10879352; doi:10.1007/s11095-023-03644-4)
Supplement: Supplementary file 1 — Supplementary file1 (DOCX 1298 KB) [file 11095_2023_3644_MOESM1_ESM.docx]

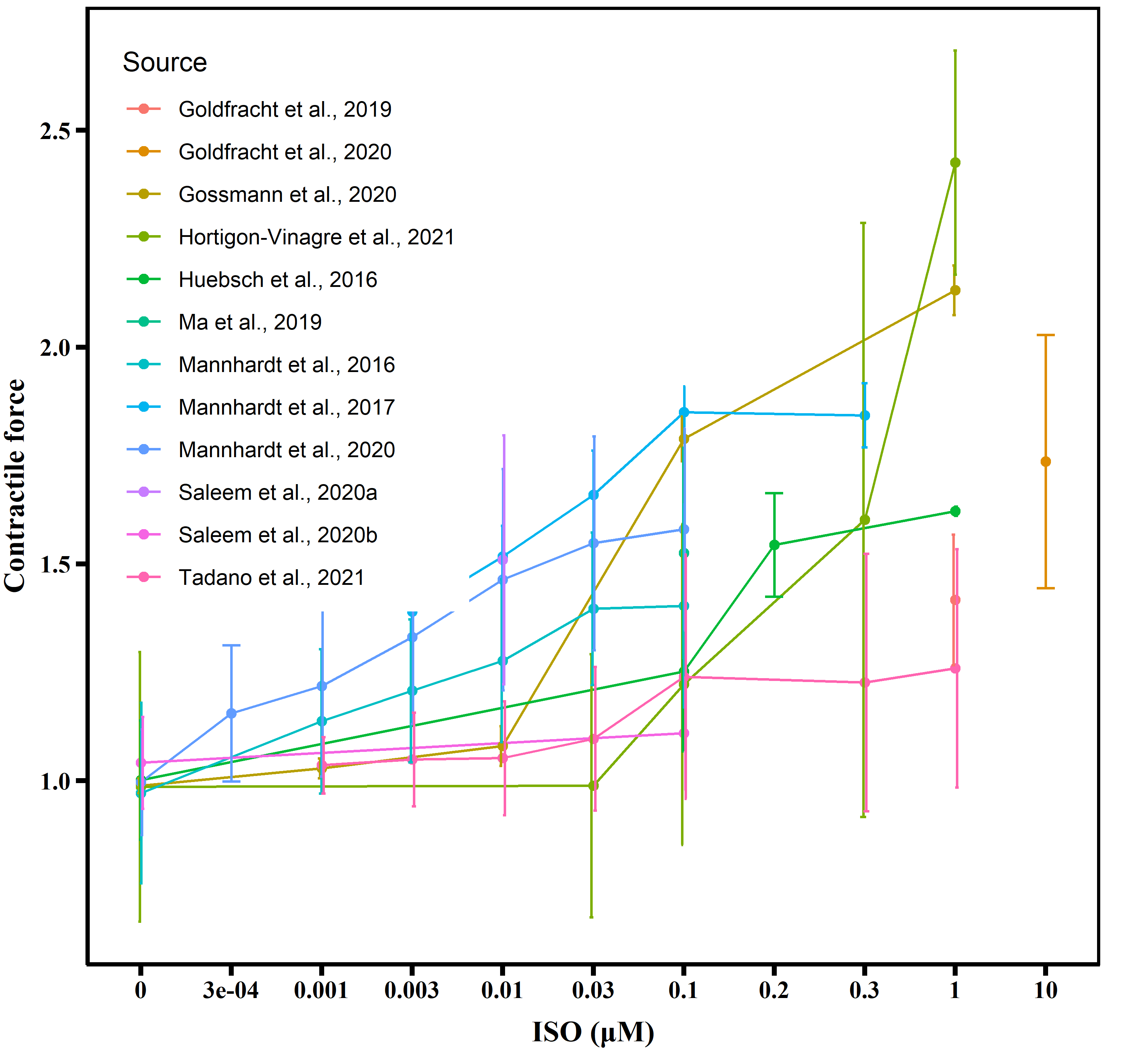


**S1 Fig. Summary of the relationship between contractile force and isoproterenol concentration.** ISO: isoproterenol.


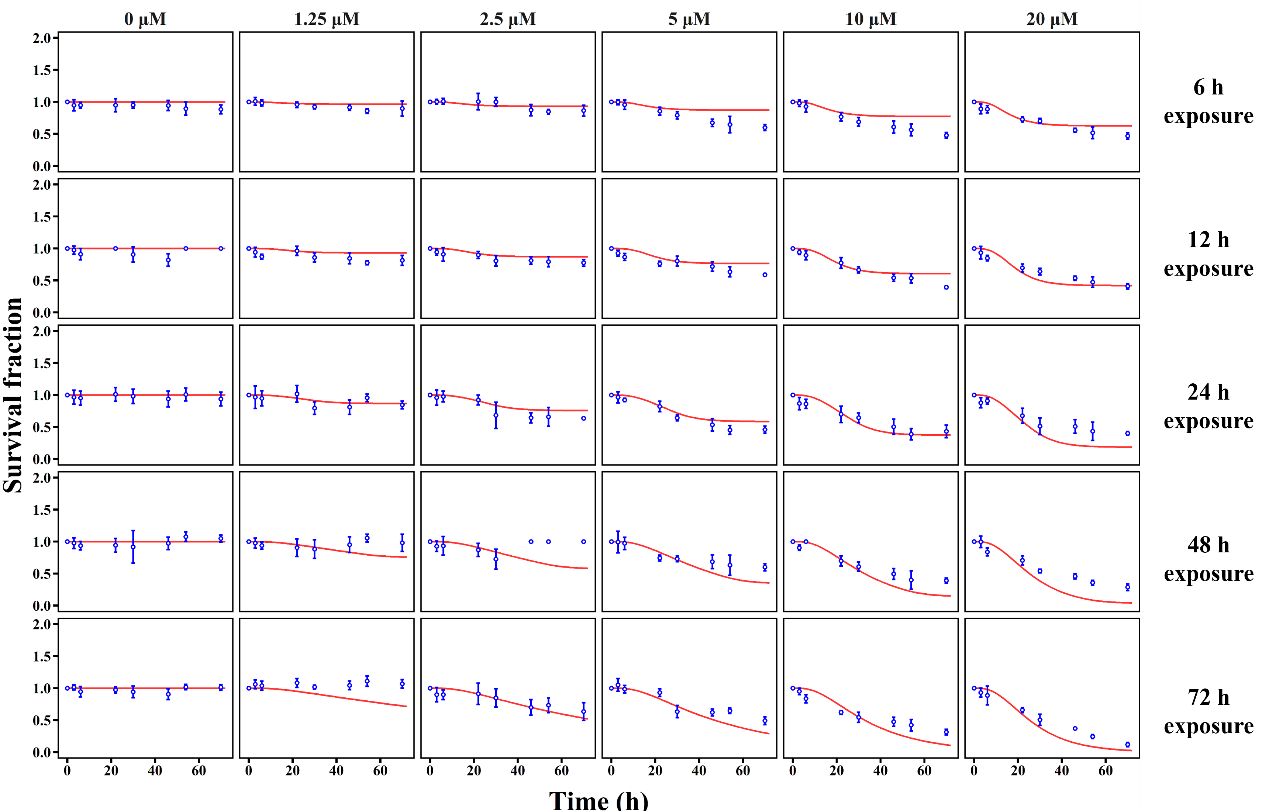


**S2 Fig. Model predictions (red line) and experimental observations (blue dot) for survival fraction in hiPSC-CMs in response to doxorubicin.** Red lines indicate model predictions and blue dots indicate experimental observations.


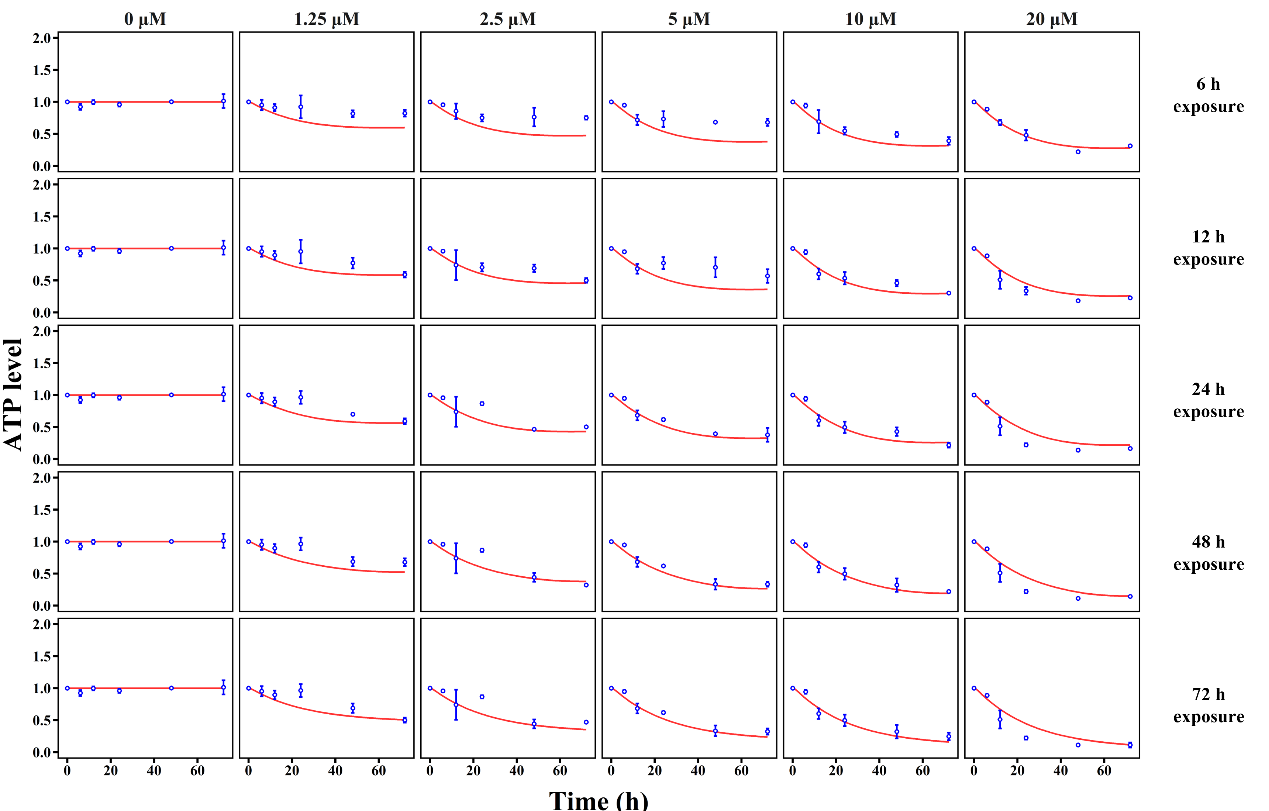


**S3 Fig. Model predictions (red line) and experimental observations (blue dot) for ATP levels in hiPSC-CMs in response to doxorubicin.** Red lines indicate model predictions and blue dots indicate experimental observations.


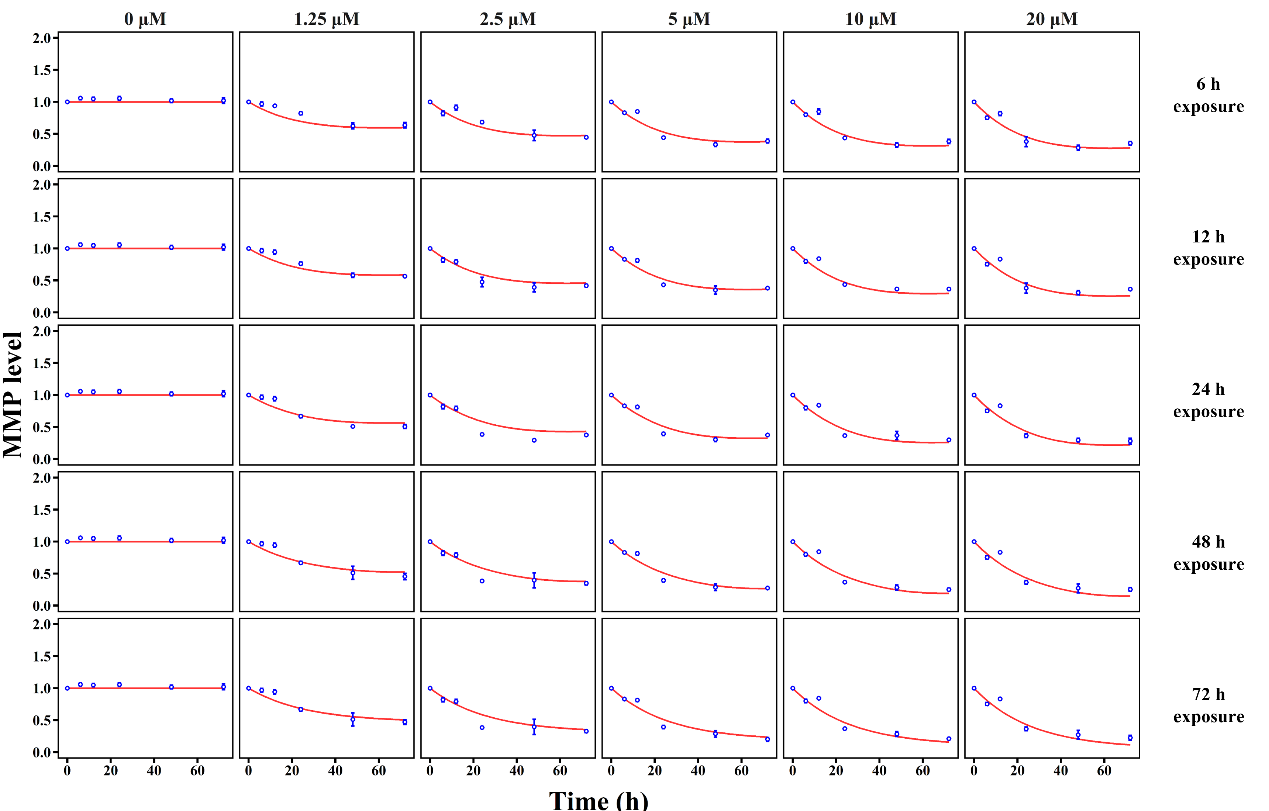


**S4 Fig. Model predictions (red line) and experimental observations (blue dot) for MMP levels in hiPSC-CMs in response to doxorubicin.** Red lines indicate model predictions and blue dots indicate experimental observations.


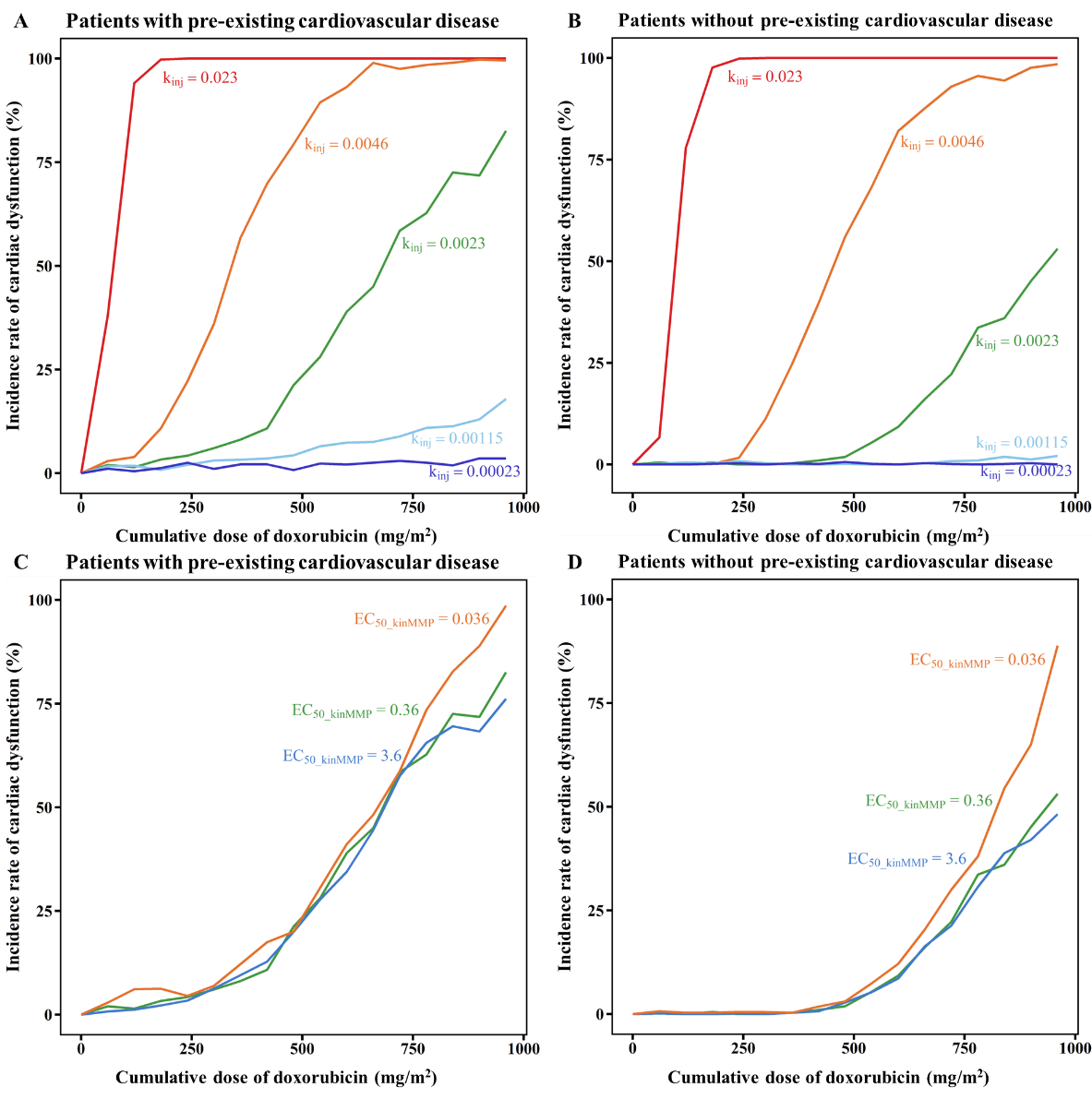


**S5 Fig. Effects of** $\boldsymbol{k}_{\boldsymbol{inj}}$ **(A, B) and** $\boldsymbol{E}\boldsymbol{C}_{\boldsymbol{50\_kinMMP}}$ **(C, D) on doxorubicin-induced cardiotoxicity in patients with (A, C) or without (B, D) pre-existing cardiovascular diseases.** $k_{inj}$: rate constant of cell injury triggered by doxorubicin; $EC_{50\_kinMMP}$: the doxorubicin concentration that cause half maximal effect on MMP production.


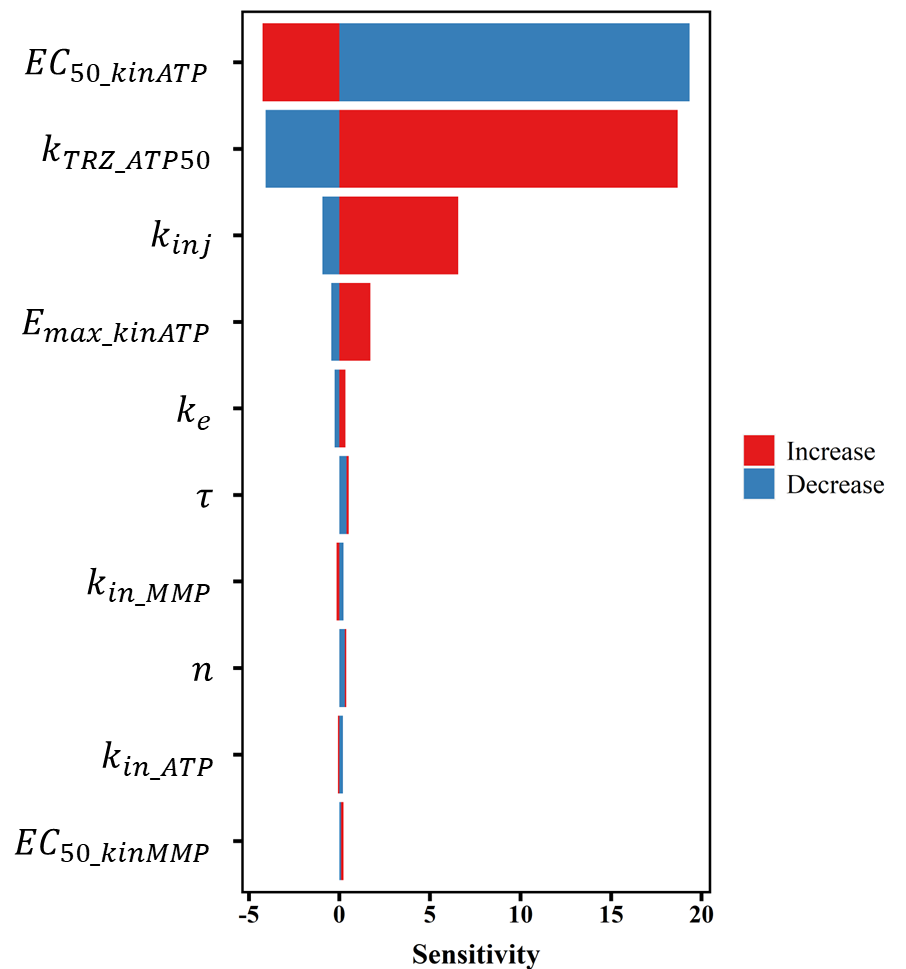


**S6 Fig. Sensitive analysis of TD parameters for trastuzumab.**


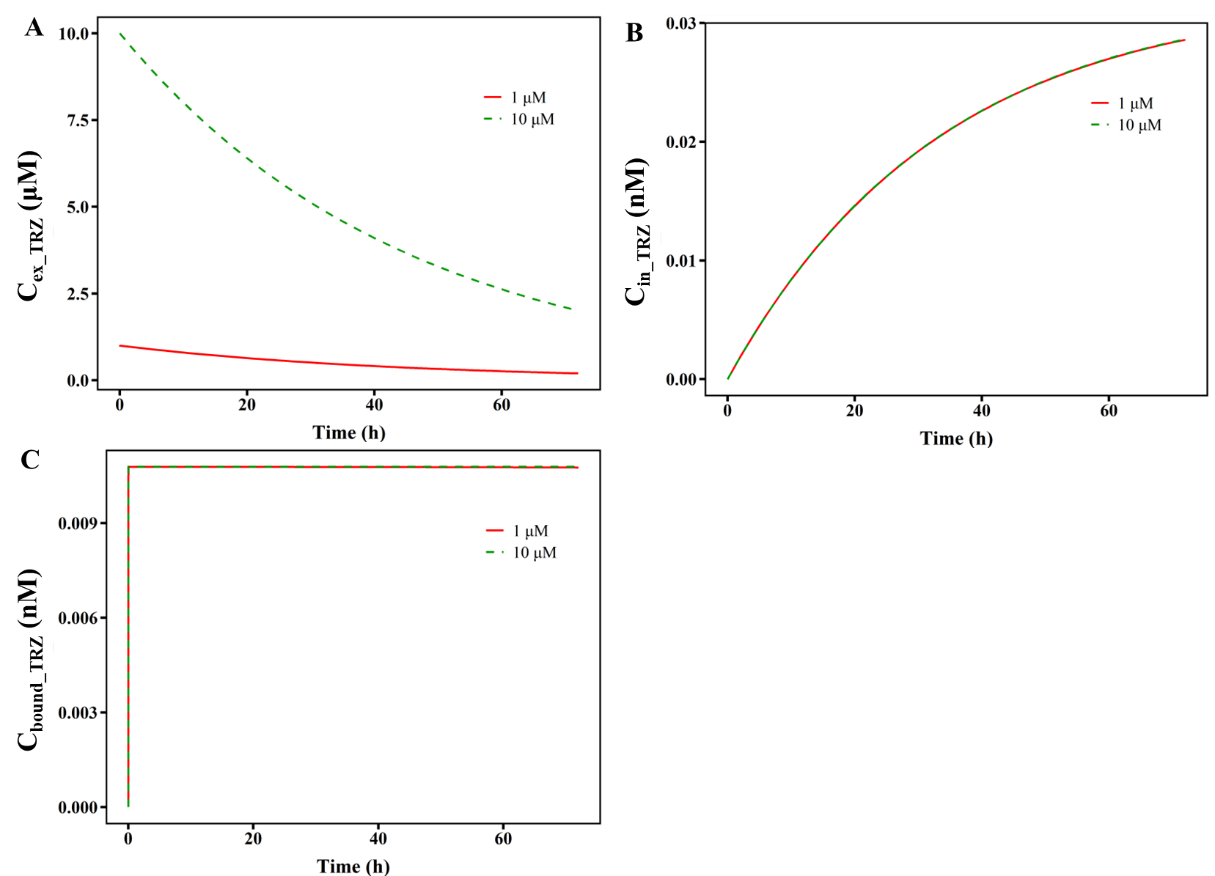


**S7 Fig. Simulated concentrations of trastuzumab.** (A) Concentration of trastuzumab in extracellular medium. (B) Concentration of trastuzumab that bind to ErbB-2. (C) Concentration of trastuzumab in intracellular fluid.

**Table S1. Explanations of TD parameters.**

| **Parameter** | **Definition** |
| --- | --- |
| $C_{in\_total}$ | Concentration of total intracellular doxorubicin |
| $C_{ex\_DOX}$ | Concentration of free doxorubicin in extracellular culture medium |
| $C_{in\_DOX}$ | Concentration of free doxorubicin in intracellular fluid |
| $PER$ | Cell membrane permeability |
| $k_{pp}$ | pH-determined difference in drug distribution between intracellular and extracellular |
| $S_{cell}$ | Surface area of a single cell |
| $CN$ | Concentration of DNA binding domain in cardiomyocytes |
| $k_{d}$ | Equilibrium dissociation constant of drug-DNA binding |
| $C_{ex\_TRZ}$ | Concentration of TRZ in the extracellular space |
| $C_{bind\_TRZ}$ | Concentration of TRZ bound to cell surface |
| $C_{in\_TRZ}$ | Concentration of internalized TRZ within the endosomal space |
| $k_{on}^{TRZ}$ | Association rate constant between TRZ and HER2 antigen |
| $k_{off}^{TRZ}$ | Dissociation rate constant between TRZ and HER2 antigen |
| $k_{int}^{TRZ}$ | Internalization rate of the TRZ inside the cell |
| $k_{deg}^{TRZ}$ | Proteasomal degradation rate of TRZ in endosomal space |
| $k_{dec}^{TRZ}$ | Non-specific deconjugation rate of TRZ from the extracellular space |
| ${Ag}_{cell}$ | Number of ErbB-2 on normal cardiomyocyte surface |
| $f_{nor}$ | Fractions of normal cardiomyocytes |
| $f_{dead}$ | Fractions of dead cardiomyocytes |
| $f_{inj}$ | Fractions of injured cardiomyocytes |
| $f_{inj\_1\sim3}$ | Fractions of injured cardiomyocytes area of single cell |
| $k_{inj}$ | Rate constant of normal cells converting to injured cells |
| $\tau$ | Transitional time of injured myocytes converting to dead cells |
| $ATP$ | Adenosine triphosphate |
| $n$ | A positive exponent for MMP’s stimulatory effect on ATP production |
| $MMP$ | Mitochondrial membrane potential |
| ${MMP}_{0}$ | The baseline MMP level, which is manually set to 1. |
| $ACF$ | Average contractile force |
| ${ACF}_{\max}$ | The maximal value of average contractile force |
| $k_{out\_ATP}$ | First-order elimination rate constants of ATP |
| $k_{in\_MMP}$ | Zero-order production rate constants of MMP |
| $k_{in\_ATP}$ | Zero-order production rate constants of ATP |
| $k_{out\_ATP}$ | First-order elimination rate constants of ATP |
| $k_{in\_MMP}$ | Zero-order production rate constants of MMP |
| $k_{out\_MMP}$ | First-order elimination rate constants of MMP |
| ${EC}_{50\_kinATP}$ | The trastuzumab concentration that cause half maximal effect on ATP production |
| $E_{max\_kinATP}$ | The maximal effect of trastuzumab on ATP production |
| $EC_{50\_kinMMP}$ | The doxorubicin concentration that causes half maximal effect on MMP production |
| $E_{max\_kinMMP}$ | The maximal effect of doxorubicin on MMP production |
| ${ATP}_{50\_ref}$ | ATP level that causes a half maximal ACF |
| ${ATP}_{50}$ | The ATP level that causes a half maximal ACF |
| $k_{TRZ\_ATP50}$ | A linear coefficient for trastuzumab |
| $k_{e}$ | The efficacy rate constant of doxorubicin’s effect |
| $E_{DOX\_TRZ}$ | The doxorubicin’s enhancement on trastuzumab’s effect on ${ATP}_{50}$ |
| $E_{drug}$ | Drug effects on myocardial contraction |
| $R_{access}$ | A ratio of effective drug concentration in cardiac interstitial fluid to that in culture medium |
